# Relationship between adolescent internet addiction and adolescent non-suicidal self-injury: a moderated mediation model

**DOI:** 10.3389/fpsyt.2024.1413167

**Published:** 2024-07-23

**Authors:** Nan Liu, Huaxiang Li, Lin Wang, Jiemei Yin, Aiqin Peng

**Affiliations:** ^1^ Nursing Department, Affiliated WuTaiShan Hospital of Medical College of Yangzhou University, Yang Zhou, China; ^2^ School of Food Science and Engineering, Yangzhou University, Yang Zhou, China

**Keywords:** IA: internet addiction, NSSI: non-suicidal self-injury, loneliness, cognitive reappraisal, adolescent

## Abstract

**Background:**

Both Internet addiction (IA) and non-suicidal self-injury (NSSI) are major public health concerns among adolescents, association between internet addiction and non-suicidal self-injury have been observed among adolescents. However, it is unclear how, and under what conditions, internet addiction relates to non-suicidal self-injury. According to our hypothesis, there is a positive relationship between IA and NSSI among Chinese adolescents, but this relationship is affected by the mediating role of loneliness and the moderating role of cognitive reappraisal.

**Method:**

A cross-sectional survey was conducted on 1046 Chinese adolescents from 3 middle schools. Measurements: Adolescent Self-Harm Scale; Young’s Internet Addiction Test (IAT); University of California at Los Angels (UCLA) Loneliness Scale; Emotional Regulation Questionnaire (ERQ), They were asked to complete self-report questionnaires.

**Results:**

In our sample, the detection rate of NSSI was 12.3%. IA was positively associated with NSSI, and loneliness partially mediated the association between them. In addition, cognitive reappraisal moderated the first half path of the mediation model. Specifically, the higher the level of cognitive reappraisal, the weaker the positive effect of IA on NSSI through loneliness.

**Conclusion:**

Interventions targeted to reduce loneliness and increase cognitive reappraisal strategies may reduce the risk of NSSI in adolescents with Internet addiction.

## 1 Introduction

Non-suicidal self-injury (NSSI) refers to behavior that causes damage to the surface of the body repeatedly in order to achieve a certain purpose without the explicit intention of suicide (1). The results of epidemiological investigation showed that NSSI behavior occurs in individuals of all age groups, with adolescents being the group with the highest frequency of occurrence (2). NSSI has become an important public health issue that significantly affects the health of adolescents globally (3). Currently, the detection rate of NSSI in China is showing a gradual upward trend. A meta-analysis of the prevalence of NSSI among adolescents in mainland China showed that the total detection rate of NSSI was 27.4% (4). This issue not only causes significant physiological and psychological harm to adolescents but also increases the risk of future suicide among individuals who engage in self-injury (5). Given the high prevalence and serious consequences of NSSI, further understanding the influencing factors and underlying mechanisms of NSSI in adolescents is necessary.

With the rapid development of the Internet, adolescents now view it as a tool that provides various opportunities for communication, education, and entertainment (6). The Internet penetration rate among minors reached 94.9% by the end of 2020, according to the data released by the China Internet Network Information Center (2021). During the COVID-19 pandemic, the Internet has become an indispensable part of adolescents’ lives, entertainment, and learning. However, while the Internet meets their increasing online entertainment and social needs, the Internet addiction (IA) is also becoming increasingly prominent (7). It is important to note that currently (2024), Internet addiction is yet to be recognized by the World Health Organization or the American Psychiatric Association as an addiction or any other disease class (8). The latest version of the International Classification of Diseases (ICD-11) includes only gaming disorder (9). However, the term has become so well-established in specialist language that it is commonly used in literature. In this paper, too, we will be using the term “Internet addiction” as a generic, non-clinical term to refer to various disorders associated with problematic Internet use. Problematic Internet use (PIU) is an umbrella construct that refers to a wide range of online behaviors (shopping, pornography viewing, social networking, cyberbullying, “cyberchondria”) that can become uncontrolled and engender negative consequences (social, occupational, familial, educational), and associated with functional impairment in a subgroup of vulnerable users (10, 11). PIU and IA are often used as synonyms (12). IA or PIU not only affects the development of adolescents’ physical health, but also endangers their mental health (13), such as insomnia, depression, anxiety. Recent studies have focused on the association of IA with NSSI (12). A European study reported that PIU among adolescents was significantly associated with self-harming/suicidal behavior (14). Empirical evidence indicated that negative emotions and inappropriate coping strategies are characteristics of IA (15) and Nock et al. (16) proposed that bad emotions and cognition combined with inappropriate coping strategies could lead to NSSI. In addition, Pan (17) showed that adolescents with IA may have neuropsychological defects, and they likely show a tendency to make risky decisions, which leads to individuals with IA adopting limited and extreme ways to cope with maladjustment or stressful events, ultimately resulting in the occurrence of NSSI. The Internet also provides convenient access to obtain information related to NSSI, promoting the occurrence of NSSI (18). According to a cross-sectional investigation in China, IA and suspicious IA behavior are independent risk factors for NSSI behavior (3). The relationship between IA and NSSI has been explored in existing research, but the results remain controversial. For example, Meszaros et al. (12) found that no direct association exists between IA and NSSI, but the relationship between the two is mediated through pathological mental disorders. Liu et al. (18) observed that after certain demographic variables were controlled, the correlation between IA and NSSI was weakened. Moreover, limited exploration has been conducted on the mechanism of action between the two. Given the harmful consequences of IA and NSSI on the healthy development of adolescents, further exploration of the mechanisms and influencing factors between these two behaviors could enhance our understanding of the trajectory and consequences of IA in adolescents.

Loneliness is a kind of negative emotion produced by an individual’s desire for interpersonal communication and intimate relationship in the social relationship network but unable to meet it (19). Several studies have shown that IA is predictive of loneliness in adolescents (20–22). Yao and Zhong (21) conducted a cross-lagged analysis and found that excessive and unhealthy Internet use increases individuals’ feelings of loneliness. Zheng et al. (22) determined through a cross-sectional study that Internet addicts experience a stronger sense of loneliness than non-Internet addicts. The Internet, which ostensibly promotes interpersonal interaction, actually makes users more distant from each other and increases their feelings of loneliness, which is called the “Internet paradox” by researchers (23). Adolescents’ internet use takes away time that might be used for social interaction. However, the relationships created and maintained through the Internet are often superficial. The substitution of lower-quality relationships through the Internet for higher-quality relationships in face-to-face interactions leads to lower levels of social involvement and increased loneliness (24). Adolescence is a high-risk period for loneliness, and loneliness is the most common of health risk behaviors among adolescents (25). Ma et al. (3) emphasized that high loneliness and low emotional management in adolescents are independent risk factors for NSSI.

The experiential avoidance model (EAM) of NSSI supposes that when an individual encounters an external stimulus that triggers the negative emotional response, the interaction between certain characteristics (such as high emotional intensity caused by the event and lack of emotion regulation strategies) of the individual and environment and other factors can prompt an individual to choose self-injury to avoid the negative experiences caused by negative emotions. Self-injury temporarily relieves their negative emotional experiences and further strengthens NSSI, which ultimately promotes the maintenance of NSSI (26). From this perspective, negative emotions are not only the result of external stimuli but also a contributing factor to hazardous behavior, which is the intermediary variable between external stimuli and behavioral consequences. On the basis of EAM, we speculate that adolescents in the period of drastic physical and mental changes with prominent rebellious psychology are prone to negative emotional experiences, such as loneliness, under the stimulation of IA. To avoid this negative emotional experience (loneliness), self-harming behavior is adopted, that is, loneliness may play a mediating role in the relationship between IA and NSSI.

Lack of effective emotion regulation strategies is one of the important factors in the development of NSSI (27). According to the model of emotion regulation process proposed by Gross (28), emotion regulation is developed in the process of emotion occurrence and development. At different stages of emotion development, there are different emotion regulation strategies. Cognitive reappraisal and expression suppression are the two most representative emotion regulation strategies (28). Both emotional regulation strategies were closely related to the occurrence and development of NSSI (29). Cognitive reappraisal is a positive emotion regulation strategy that generally occurs before the emotional response (28), that is, by changing the cognition and understanding of emotional events (e.g. interpreting a remark as benign/neutral instead of insulting/personal), individuals reduce the negative emotions associated with situations to reduce their negative emotions and psychological pain (30). It belongs to antecedent-focused emotion regulation strategy. Cognitive reappraisal strategies could help individuals understand the events that cause negative emotions in a positive way to reduce the experience of negative emotions and the expression of negative behaviors (e.g., self-harm) (31, 32). Evidence suggested that cognitive reappraisal played a significant protective role in self-harm because individuals who employ cognitive reappraisal could effectively reduce the risk and severity of self-injury (31, 33). A 3-year longitudinal study found that cognitive reappraisal was still significantly negatively associated with NSSI while controlling for negative life events, psychological stress, and other ways of emotional regulation (34). However, expression suppression, a response-focused emotion regulation strategy, generally occurs after emotions have been formed or the emotional response is activated. It suppresses individuals’ emotional expression by mobilizing their self-control ability (e.g. when someone steps on your foot and he doesn’t apologize, you try to control your anger even though you are angry). Although expression suppression could reduce individuals’ emotional expression, it does not reduce the individuals’ psychological experience of emotion (35). It is a significant positive predictor of adolescent NSSI behavior (36).

Therefore, cognitive reappraisal may weaken the association between IA (emotional situation) and loneliness (negative emotional response to the situation) when a situation may cause emotional fluctuations of individuals (IA). That is, individuals with high levels of cognitive reappraisal may adjust their cognition and understanding of the current situation to change the consequent negative emotional (loneliness) effect and increase the experience and expression of positive emotions, thereby reducing the occurrence of NSSI. When the emotional response is activated, individuals try to suppress the negative emotion using expressive suppression. However, this inhibition may paradoxically promote the lingering and accumulation of negative emotions, which exacerbates negative psychological experiences and thus increases the risk of NSSI.

The cognitive-emotional model of NSSI (1) further supports the role of emotion regulation played by cognitive reappraisal in the NSSI. This model proposes that emotion and cognition can work in concert to govern NSSI. Cognitive reappraisal, as an adaptive emotion regulation strategy, changes the trajectory of emotional responses by reinterpreting emotional events, which may weaken the association between a perceived emotionally volatile situation and a person’ s emotional response to it, ultimately reducing self-harming behaviors.

On the basis this model, Gu et al. (33) found that the direct impact of harsh parenting on adolescents’ self-injurious behavior and the indirect impact of alienation are moderated by cognitive reappraisal. In other words, adolescents with high cognitive reappraisal level have lower feelings of helplessness, loneliness, and other alienation even though they are subjected to high levels of harsh parenting. They are less likely to have self-harming behaviors. Another related study by Gu et al. (37) showed that the indirect effect of self-criticism on NSSI through psychological pain is moderated by cognitive reassessment, which can weaken the indirect effect of self-criticism on NSSI.

Based on the above theoretical model and previous research results, cognitive reappraisal generally occurs in the early stage of emotion generation, and has a significant easing effect on negative emotions caused by emotional situations. We supposed that cognitive reappraisal should be able to moderate the mediating (loneliness) process by weakening the relationship between IA and loneliness, namely, the first paragraph of the mediation pathway.

Based on these findings, we hypothesized the following:

H1. Adolescent IA is positively associated with NSSI.

H2. Loneliness will mediate the relationship between IA and NSSI.

**Figure 1:**
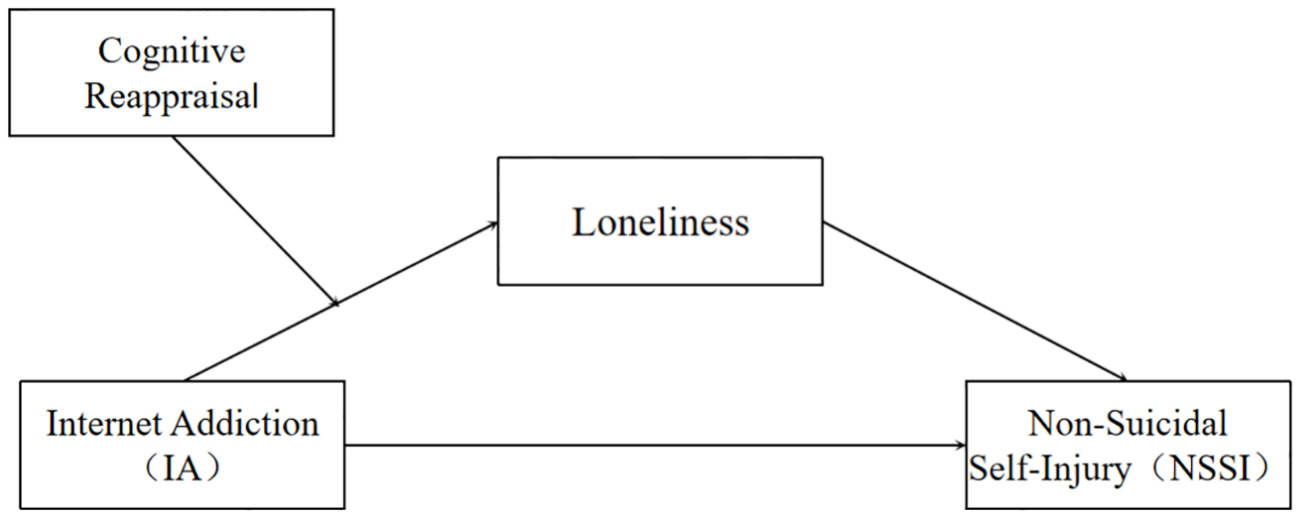
A moderated mediation model of Internet Addiction (IA), Loneliness, Cognitive Reappraisal, and Non-Suicidal Self-Injury (NSSI).

H3. The mediating role of loneliness between IA and NSSI will be moderated by cognitive reappraisal. Specifically, cognitive reappraisal negatively moderates the first half of the mediating model (**Figure 1**).

The findings of this study will contribute to understanding the relationships between IA, loneliness, cognitive reappraisal, and NSSI. This understanding could help in timely interventions to prevent further negative behaviors among adolescents. At the same time, it would provide ideas for formulating intervention strategies to reduce NSSI behavior, which in turn would decrease the occurrence of negative events and help adolescents grow up healthily.

## 2 Materials and methods

### 2.1 Participants

In this study, 1118 students from 3 middle schools in Yangzhou, Jiangsu Province were investigated using a convenience sampling method. The three secondary schools include a general middle school, a general high school, and a vocational high school (vocational high school is a secondary school that combines ordinary high school education with vocational education. It not only provides students with general high school education but also implements vocational knowledge and skill education in accordance with the requirements of vocational positions).

### 2.2 Procedures

First, the researchers involved in the study were trained uniformly. In the selected secondary schools, group testing was conducted on a class basis. Prior to data collection, students and their parents were invited to attend a parent-teacher conference where the researchers would explain the purpose, method, and significance of this study. Then, the researchers would ask students and their parents about their willingness to participate in the study and inform them that they could voluntarily choose whether to take part in this study or not. After the permission of students and their parents, they signed written informed consent. Secondly, under the supervision and guidance of trained researchers, students filled out self-reported questionnaires on demographics, IA, loneliness, cognitive reappraisal, and NSSI in an organized classroom setting, and students were told that the questionnaire was anonymous. Finally, after students completed the questionnaire, the researchers collected the questionnaire on the spot. The questionnaires took about 20 minutes to complete. No reward or compensation was given. Seventy two of these participants were excluded from the sample because they showed an obvious pattern of response (e.g., selecting “1” for all items). The final valid questionnaires were 1046.

### 2.3 Measure

#### 2.3.1 Non-suicidal self-injury

NSSI was assessed using the Adolescent Self-Harm Scale, which was developed by Zheng (38) and revised by Feng (39). The scale measures the frequency and severity of 18 kinds of NSSI, such as cutting, burning, stabbing, and rope wound, in the past year. Participants rated how often these items occurred on a 0–3 scale (0 = never, 1 time = 1, 2–4 times = 2, 5 or more times = 3). At the same time, they rated the severity with which these items occurred on a 0–4 scale (0 = none, mild = 1, moderate = 2, severe = 3, extremely severe = 4). The level of NSSI was assessed using the composite score, which was calculated by multiplying the frequency of self-harming incidents with the severity of physical harm. A higher score indicated a more severe level of NSSI. In this study, Cronbach’s *α* for this scale was 0.754.

#### 2.3.2 Internet addiction

Young’s Internet Addiction Test (ITA) (40) was used to assess adolescent Internet addiction. The scale comprises 20 items using a 5–point Likert scale (1 = rarely; 2 = occasionally; 3 = frequently; 4 = often; 5 = always). The total scores ranges from 20 to 100. Three types of internet user groups were identified based on the original cutoff points proposed by Young, namely “average online users” (20–49 points), “moderate IA” (50–79 points), and “severe IA” (80–100 points). In this study, Cronbach’s *α* for this scale was 0.982.

#### 2.3.3 Loneliness

The UCLA Loneliness Scale (UCLA Loneliness Scale, University of California at Los Angels) revised by Russell et al. (41) was used to assess adolescents’ loneliness. The scale comprises 20 items using a 4–point Likert scale (1 = never; 2 = rarely; 3 = often; 4 = always). Higher scores indicated higher levels of loneliness. Based on the actual scores, loneliness levels were divided accordingly. Scores below 28 indicated low levels of loneliness, scores ranging from 18 to 33 indicated mild to moderate levels of loneliness, scores between 33 and 39 indicated moderate levels of loneliness, scores from 39 to 44 indicated moderate to high levels of loneliness, and scores above 44 indicated high levels of loneliness. In the present study, Cronbach’s α for this scale was 0.959.

#### 2.3.4 Cognitive reappraisal

Cognitive reappraisal was assessed using the cognitive reappraisal subscale of the Emotional Regulation Questionnaire (28). The scale comprises six items using a 5–point Likert scale (1 = strongly disagree; 5 = strongly agree). Higher scores indicated higher levels of cognitive reappraisal. A Chinese version of the scale (42) was used in the present study, and Cronbach’s *α* for this scale was 0.943.

#### 2.3.5 Control variables

Previous studies have shown that NSSI in adolescents is significantly correlated with gender and age (3, 43). Hence, we included these variables as control variables in the statistical analysis.

### 2.4 Data analysis

The data were analyzed using the SPSS 23.0 software and PROCESS macro 3.3 (44).

First, statistical descriptions of demographic data and study variables were presented using M (means) ± SD (standard deviations), rate or component ratio. NSSI scores were performed in univariate analysis in different demographic variables, independent sample t-test or one-way ANOVA and non-parametric test (Kruskal-Wallis H test) were used according to the normal distribution and/or homogeneity of variance of the data. Pearson correlations among variables were calculated. Second, following the two preliminary data analyses, PROCESS Model 4 (44) was used to test the mediation of loneliness. The bias-corrected bootstrapping method based on 5000 samples was used to test the significance of the indirect effect, which was regarded as significant if the 95% confidence interval (CI) did not include zero. Third, the moderated mediation was analyzed using PROCESS Model 7 (44). All continuous variables were normalized, and interaction terms were calculated on the basis of these normalized scores. Finally, a simple slope analysis was used to test whether the mediation effect of loneliness differed at various levels of the moderator variable. In addition, we used the Johnson-Neyman technique to determine in which regions of cognitive reappraisal the effect of IA on loneliness is significant and non-significant.

### 2.5 Common method bias

This study followed a previous method proposed to regulate the deviation of common methods (such as anonymous method and reverse scoring) to control the testing process (45). The common method bias was tested by Harman’s single-factor test. The results showed that 13 factors had feature roots greater than 1, and the explanation rate of the first common factor was 30.55%, which was less than the critical standard of 40% (46). Hence, no serious common method bias occurred in this study.

## 3 Results

### 3.1 Descriptive analysis

The demographic data of 1046 participants were displayed in **Tables 1**, **2**. The participants ranged in age from 12 to 18 years (M = 15.69, SD = 1.594), of whom 57.6% (n = 603) were male. The proportion of students in grades 7–9 was 10.8% (n = 113), 15.7% (n = 164), and 12.8% (n = 134), respectively. The proportion of students in general high schools was 13.9% (n = 145), 9.1% (n = 95), and 7.8% (n = 82), while the proportion of students in vocational high schools was 10.2% (n = 107), 10.3% (n = 108), and 9.4% (n = 98). Of the participants, 49% (n = 513) were single child family, and 79.9% (n = 836) reported that their place of residence was urban, 54.4% (n = 569) of families are nuclear families, 71.7% (n = 750) of the participants self-perceived the family atmosphere as good, and 49.24% of the participants considered their family economic conditions to be average.

**Table 1:** Demographicl Information of samples (n=1046).

Factors	Subject	Number	Percent(%)	*M*	*SD*	*t/F*	*p*
Gender	Male	603	57.65	1.542	6.249	-1.667	0.096
	Female	443	42.35	2.368	8.941		
Age	−	1046	−	15.69	1.594	−	−
Residence	Urban	836	79.92	1.623	6.996	1.963	0.051
	Rural	210	20.08	2.962	9.241		
Family Structure	Single-parent family/Reorganized family	74	7.07	2.662	8.992		
	Extended family	403	38.53	1.551	6.324		
	Nuclear family	569	54.40	2.033	8.064	0.904	0.405
Perceived family atmosphere	Poor	30	2.87	1.633	6.234		
	General	266	25.43	2.286	7.653		
	Good	750	71.70	1.763	7.517	0.493	0.611
Family economic conditions	Poor	67	6.4	1.164	3.776	0.562	0.571
	Average	515	49.24	1.851	7.316		
	Good	307	29.35	2.205	7.986		
	Superior	157	15.01	1.726	8.390	0.417	0.741
Single child	Yes	513	49.04	1.950	8.274		
	No	533	50.96	1.837	6.712	0.242	0.809

M, Mean; SD, Standard deviations.

**Table 2:** Demographicl Information of samples (n=1046).

	Factors	Subject	Number	Percent(%)	*M*	*SD*	*z/x2*	*p*
School nature	General middle school	Grade7	113	10.8%	1.673	4.034		
		Grade8	164	15.7%	0.165	0.888		
		Grade9	134	12.8%	8.403	16.216		
	General high school	Grade10	145	13.9%	3.041	9.169		
		Grade11	95	9.1%	< 0.001	< 0.001		
		Grade12	82	7.8%	< 0.001	< 0.001		
	Vocational high school	Grade10	107	10.2%	1.234	3.791		
		Grade11	108	10.3%	0.593	2.881		
		Grade12	98	9.4%	< 0.001	< 0.001	124.241	< 0.001

M, Mean; SD, Standard deviations.

In terms of the Adolescent Self-Harm Scale, the total score of 1,046 participants was 1.89 ± 7.515. 12.3% of participants (n = 129) reported at least one incidence of NSSI in the previous 12 months.

### 3.2 Preliminary analyses

**Table 3** indicates the descriptive statistics of related variables, including Mean (*M*), standard deviations (*SD*), and the bivariate correlation analysis between above variables. After controlling for two general demographic variables, gender and age, the results of correlation analysis showed that IA, loneliness and NSSI were significantly positively correlated with each other (*r* = 0.290 ∼ 0.476, *p* < 0. 01), and cognitive reappraisal was negatively correlated with these variables (| *r* | = 0. 195 ∼ 0. 494, *p* < 0. 01). The prima facie evidence for the assumed moderated mediation model was offered by the findings of the above correlation analysis.

**Table 3:** Descriptive statistics and correlation analysis of variables.

	*M*	*SD*	1	2	3	4
1.IA	41.39	20.993	1			
2.CR	23.05	11.942	-0.253**	1		
3.Loneliness	43.30	16.568	0.290**	-0.494**	1	
4.NSSI	1.89	7.515	0.476**	-0.195**	0.378**	1

IA, Internet addiction; CR, Cognitive reappraisal; NSSI, non-suicidal self-injury.

M, Mean; SD, Standard deviations.

N=1046; **p < 0.01.

### 3.3 Tests of mediating effect of loneliness

According to Hayes (44), we tested the mediating effect of loneliness on IA and NSSI. The mediating role of loneliness was upheld. After controlling for gender and age, IA positively affected NSSI, c = 0.171, *p* < 0.001; then, IA and loneliness entered the regression equation at the same time, the result showed that IA positively affected loneliness, a = 0.245, *p* < 0.001; loneliness positively affected NSSI, B= 0.122, *p* < 0.001; IA positively affected NSSI, c’ = 0.141, *p* < 0.001 (see **Table 4** for details). Finally, the bias corrected percentile Bootstrap method test showed that loneliness play a significant mediated role between IA and NSSI, *ab* = 0.030, *SE* = 0.005, and 95% CI = [0.021, 0.040] (see **Table 5** for details). Thus, loneliness partially mediated the relationship between IA and NSSI. The mediation effect accounts for 18% of the total effect. Therefore, Hypotheses 1 and 2 were validated.

**Table 4:** The moderated-mediating effect of IA on NSSI.

Dependent variable	Model 1 (NSSI)		Model 2 (Loneliness)		Model 3 (NSSI)		Model 4 (Loneliness)	
	β[95% CI]	*t*	β[95% CI]	*t*	β[95% CI]	*t*	β[95% CI]	*t*
Gender	0.138(-0.746 to 1.022)	0.307	-3.831(-5.927 to -1.735)	-3.586**	0.606(-0.246 to 1.458)	1.396	-3.804(-5.537 to -2.072)	-4.308***
Age	0.012(-0.261 to 0.285)	0.087	-1.511(-2.159 to -0.863)	-4.576***	0.197(-0.068 to 0.461)	1.459	-1.325(-1.893 to -0.757)	-4.575***
IA	0.171(0.151 to 0.190)	17.278***	0.245(0.199 to 0.291)	10.452***	0.141 (0.121 to 0.160	14.159***	0.152(0.106 to 0.199)	6.453***
Loneliness					0.122(0.098 to 0.147)	9.759***		
CR							-0.565(-0.660 to -0.470)	-11.677***
IA×CR							-0.010(-0.013 to -0.006)	-4.967***
R^2^	0.227		0.105		0.292		0.316	
F	101.999***		40.896***		107.226***		142.800***	

IA, Internet addiction; CR, Cognitive reappraisal; NSSI, Non-Suicidal Self-Injury.

N=1046; Gender was coded as 0 = male and 1 = female. **p < 0.01; ***p < 0.001.

**Table 5:** The Bootstrapping analysis of the mediating effects.

	Effect	SE	Boot CI lower	Boot CI upper	Proportion
Total effect	0.171	0.010	0.151	0.190	
Direct effect	0.141	0.010	0.121	0.160	82%
Indirect effect	0.030	0.005	0.021	0.040	18%

N=1046.

CI = 95 % confidence interval.

### 3.4 Test of moderated mediation model effect

Model 7 of PROCESS macro 3.3 was used to test the moderating effect of cognitive reappraisal. The results are shown in **Table 4**, IA had a positive predictive effect on loneliness(*β* = 0.152, *p* < 0.001), Cognitive reappraisal has a negative predictive effect on loneliness (*β* = -0.565, *p* < 0.001). The interaction between Internet addiction and cognitive reappraisal significantly predicted loneliness (*β* = -0.010, *p* < 0.001), and the index of the moderated mediation was -0.0012, SE = 0.0003, 95% CI = [-0.0018, -0.0007], suggesting cognitive reappraisal moderated the association between IA and loneliness.

**Figure 2:**
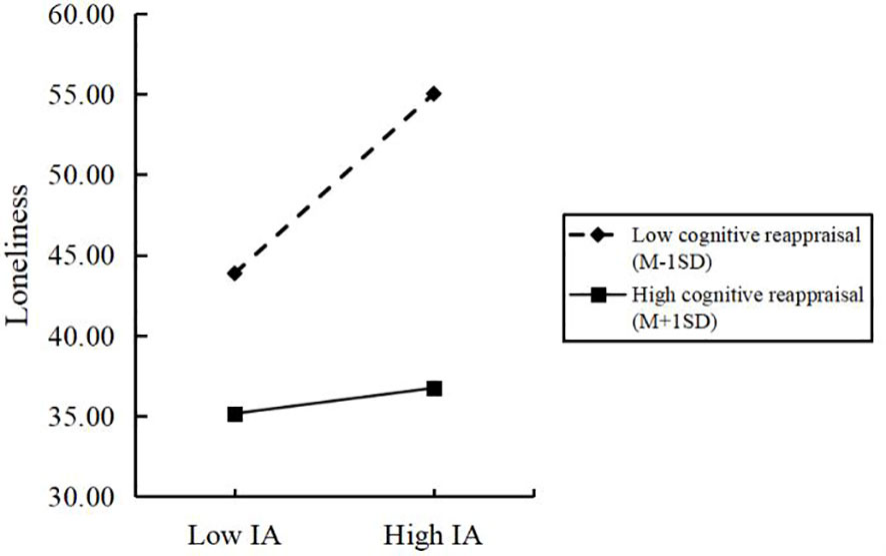
Cognitve reappraisal as a moderator on the relationship between internet addiction and loneliness. IA, Internet addiction.

To clarify the essence of the interaction effect between IA and loneliness, we divided cognitive reappraisal into high and low groups in accordance with the average plus or minus an *SD* and used a simple slope analysis to explore the role of cognitive reappraisal in the relationship between IA and loneliness. The specific moderating effect is shown in **Figure 2**. When the level of cognitive reappraisal was low, IA had a significant positive predictive effect on loneliness (Bsimple = 0.267, SE = 0. 027, *p* < 0. 001, 95% CI = [0.214, 0.319]). However, when the level of cognitive reappraisal was high, IA had no significant predictive effect on loneliness (Bsimple = 0.038, SE = 0. 038, *p* = 0.319, 95% CI = [−0.037, 0.113]). The indirect effect of loneliness on adolescents with low cognitive reappraisal level (M − 1SD) was significant (index = 0.033, Boot SE = 0.005, 95% CI = [0.023, 0.044]), whereas the indirect effect on adolescents with high cognitive reappraisal level (M + 1SD) was in significant (index = 0.005, Boot SE = 0.005, 95% CI = [−0.004, 0.014]), as shown in **Table 6**. Thus, we concluded that the mediating effect of loneliness decreased significantly with the increasing level of cognitive reappraisal, which plays a buffering role in the indirect effects of IA on NSSI.

**Table 6:** Conditional indirect effect of cognitive reappraisal when loneliness mediated between IA and NSSI.

Mediator	cognitive reappraisal	Effect	BootSE	BootLLCI	BootULCI
Loneliness	M − 1SD	0.033	0.005	0.023	0.044
	M	0.019	0.004	0.012	0.027
	M+ 1SD	0.005	0.005	-0.004	0.014

N=1046.

CI, 95 % confidence interval; LL, low limit; UL, upper limit.

**Figure 3:**
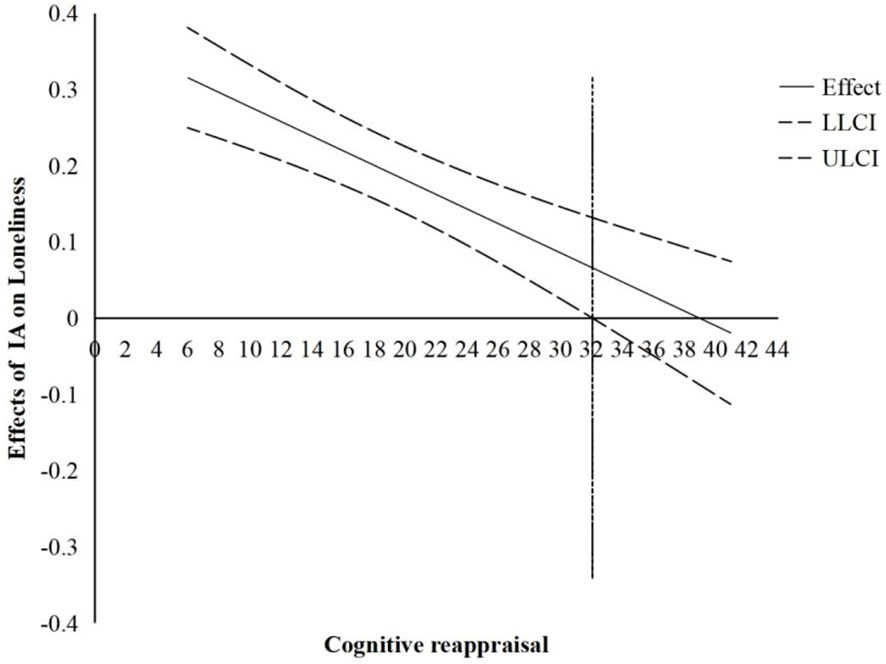
The conditional effect of IA on Loneliness as linear function of cognitive reappraisal. IA, Internet addiction; CI, 95% confidence interval; LL, low limit; UL, upper limit.

Furthermore, we used the Johnson-Neyman technique to identify the regions in the range of the moderator variable where the effect of the IA on loneliness is statistically significant and not significant (47, 48). The results may help provide more preventive interventions and pointed suggestions. As shown in **Figure 3**, the positive correlation between IA and loneliness was significant when the cognitive reappraisal value was less than 32, and the association was relatively stronger for adolescents who had lower cognitive reappraisal. However, when the cognitive reappraisal value was higher than 32, the effect of IA on loneliness was not significant.

Overall, these results suggested that cognitive reappraisal moderated the relationship between IA and NSSI via loneliness. Hypothesis 3 was supported.

## 4 Discussion

IA and NSSI are serious public health problems that endanger the physical and mental health of adolescents. In this study, the moderating mediation model was used to reveal the relationship between IA and NSSI and to clarify the underlying predictive mechanism of IA to NSSI. First, it illustrated how IA affects NSSI, including its direct effects and the mediating role of loneliness. Second, it analyzed “how it works further.” In other words, the mediating effect of loneliness was distinguished as being influenced by different levels of cognitive reappraisal.

According to our survey, the detection rate of NSSI among participants was 10.6%, which was lower than the results of previous studies on middle school students (49), but was still within the range of detection rates of 5.4% – 23.2% obtained by domestic studies (50). The variation was probably caused by different researchers’ criteria for defining NSSI and the different measurement tools used in studies. Nevertheless, these findings reminded us that NSSI cannot be ignored among middle school students.

In our study, the results showed a significant positive correlation between IA and NSSI. Specifically, the higher the level of IA, the higher the likelihood of NSSI, which supported Hypothesis 1. These results were consistent with the results of most previous studies (51, 52).

As for the reasons why IA affects NSSI, Liu et al. (18) believed that the deindividuation caused by IA affected adolescents’ normal social function and reduced their emotions such as alertness and fear. They were prone to impulse control disorder, which is characterized by repetitive and uncontrollable actions harmful to oneself or others. This phenomenon leads to impulsive behaviors, such as self-injury. The Internet provides a way for NSSI; with the increasing use of the Internet in our daily lives, more interactive online activities provide adolescents with social networking opportunities that are not restricted by traditional physical boundaries or monitored by adults. Adolescents addicted to the Internet likely obtain information about NSSI through relevant websites and forums, thus promoting the occurrence of NSSI (18). In addition, adolescents with IA tend to show decreased confidence and poor ability to resist pressure in the face of frustration and bad emotions (53). These characteristics provide a prerequisite for adolescents to develop NSSI behaviors (53). Therefore, we should take corresponding measures to protect teenagers against IA. Effective control of adolescents’ Internet use may reduce the risk of NSSI and promote the healthy development of adolescents. For example, exercise intervention, several studies have pointed out that moderate-intensity physical exercise (54) and high-intensity interval exercise (55) can relieve the symptoms of IA. The forms of movement mainly include a single sports form and “sports +” two types. The single sports form mainly includes three types: Group confrontation sports (e.g., football and basketball) (56, 57); Aerobic exercise (e.g., Tai Chi) (58); Leisure sports (e.g., sports games, outward bound training) (59). The intervention form of “sports +” mainly includes the combination of sports and psychological counseling (60).

In addition to the direct effect of IA on NSSI, this study also found that IA can indirectly affect adolescents’ NSSI through loneliness. Some adolescents with IA do not directly exhibit NSSI, but rather a sense of loneliness that is associated with an increased risk of NSSI. Hypothesis 2 was verified. Therefore, the increase in loneliness could be used as a mechanism to explain the relationship between IA and NSSI in adolescents. Adolescents with IA indulge in the virtual world for a long time, which greatly reduces their face-to-face social time with their families or peers. However, most network communications have the characteristics of surface and weak connection strength (61). Weak social bonds online are not enough to replace offline socializing, nor can they provide adequate emotional support, leading to a rising sense of loneliness among them (62). In China, students’ academic performance is highly valued. Students with high academic achievement are often regarded as successful, so they are more likely to be respected by their peers and have a higher status in their peers; by contrast, students with poor academic performance tend to have a certain disadvantage in peer relationships (63) and have a lower social status in their peers (64). Some studies have shown that many students with IA have low academic achievement. In real life, they tend to experience culturally defined failures (65), which makes them likely feel inadequate social support (12) and leads to increased loneliness. Low academic achievement also tends to make them feel inferior (66), which increases the tendency of social avoidance in the real society (67) and the negativity toward social relations (20). Consequently, they hardly achieve satisfaction in normal social interactions, which aggravates their loneliness (20).

According to the two-dimensional four-function model proposed by Nock (16), self-negative reinforcement is the most common function of NSSI, that is, NSSI is the management and regulation of negative emotions (such as loneliness) to obtain emotional relief and balance. Applied to our study, NSSI is a form of management and regulation of negative emotions such as loneliness, wherein loneliness caused by IA can in turn lead to NSSI as a means to reduce emotional pain. In addition, adolescents with a strong sense of loneliness are usually accompanied with low self-esteem and low self-evaluation and are prone to choosing extreme ways to cope with things (such as self-injurious behavior) (68). Therefore, interventions that focus on alleviating loneliness in adolescents may be a potential strategy for preventing NSSI. Peer support is one of the main sources of social support for adolescents. Several studies have shown that friendship quality is strongly associated with loneliness in adolescents (69). Zhou (70) conducted focused group counseling for junior middle school students with the theme of “Friendship Quality”, which improved the quality of friendship among them and alleviated their loneliness. Therefore, schools should not only pay attention to students’ academic achievements, but also pay attention to the cultivation of good peer relationships among classmates. Teachers should establish good teacher-student relationship with teenagers and improve their own teacher justice quality. Teachers’ justice quality includes treating students equally, treating them equally, teaching them according to their aptitude, and rationally distributing the educational resources they have in specific educational activities (71). Research has shown that teacher justice can help students get more teacher support and peer support, which can reduce adolescents’ experience of loneliness and ultimately avoid the occurrence of self-harming behaviors (72). Family is a place for teenagers to relax and relax. Parents could improve co-parenting awareness and skills to reduce parental conflict, thereby reducing loneliness and depression symptoms, and preventing NSSI in adolescents (73).

This study confirmed that cognitive reappraisal moderated the indirect role of loneliness in the association of IA and NSSI. Its regulatory effect occurred in the first half of the mediation pathway.

Specifically, at a low cognitive reappraisal level (cognitive reappraisal value was less than 32), the positive effect of IA on loneliness was enhanced; at a high cognitive reappraisal level, IA and loneliness were uncorrelated. In other words, a higher level of cognitive reappraisal could serve as a protective factor for NSSI, buffering the effect of IA on NSSI through loneliness. This finding is consistent with that of previous research (33), which showed that in the context of emotional stimuli, adolescents with low cognitive reappraisal ability were likely to experience negative emotions and were inclined to relieving these negative emotions through self-injury.

Gross on the basis of the emotional regulation process model, proposed that cognitive reappraisal is a process in which individuals reduce their emotional responses by changing their understanding of emotional events. The study has shown that adolescents with high cognitive reappraisal have good interpersonal functioning and social adjustment (28).

They can review the relationship between the Internet and social satisfaction and then change their cognition that online virtual socialization is the main social way to meet their social needs, properly use Internet tools to maintain and expand social relations in reality, diversify their social avenues, take the initiative to increase opportunities for face-to-face communication and interaction, and gain genuine emotional support. In this way, the alienation of interpersonal relationship caused by IA and the loneliness caused by the reduction in social reality can be alleviated.

Fritz (74) argued that the frequent use of cognitive reappraisal strategies can help people not only identify the positive aspects of stressors but also show other forms of positive thinking. Long-term addiction to the Internet has a great impact on teenagers’ studies, who tend to have low academic achievement. On the contrary, adolescents who are good at applying cognitive reappraisal as an emotion regulation strategy may reinterpret low academic achievement (possible emotional stimulation situations) and improve their self-awareness in interpersonal communication (75). Instead of associating low academic achievement with low social status among peers and low self-esteem, they may take the initiative to increase communication with peers and seek possible social support systems. This approach helps them gain positive feedback and a sense of belonging, which reduces their loneliness, ultimately reducing the probability of NSSI occurring. Therefore, improving cognitive reappraisal skills in adolescents may be particularly useful for the treatment of NSSI (34). For example, Bentley et al. (76) found that a cognitive reappraisal intervention effectively reduced the urges and acts of NSSI.

## 5 Limitations

This study exhibits several limitations. First, the conclusions of this research were based on the analysis of data, and the collection of data was based on self-report. Therefore, the results might be limited by social desirability and recall bias. In the future, the impact of IA on NSSI could be further explored by integrating various data collection approaches. Second, this research adopted a cross-sectional research design. The tracking research method could be applied in the future to reveal the relationship between variables in depth. In addition, this research found that loneliness played a partial mediating role in the relationship between IA and NSSI. There may be other mediating variables in the relationship between the two, so the comprehensive influence of multiple mediating variables could be considered in the future. Third, in this study, only “gender and age” were used as control variables, and other potential confounders such as school nature, socioeconomic status, and family dynamics were not considered. In future studies, we will combine existing literature and research results, and fully consider potential confounding factors that may exist to strengthen the validity of the study. Lastly, the participants in this study were from Yangzhou, China; whether our findings could be generalized to adolescents from other regions of China or other cultures needs to be tested in the future.

## 6 Conclusion

This research identified a significant moderated mediation model through cross-sectional design that explained the effect of IA on NSSI in adolescents. Our findings suggest that IA is associated with an increased risk for adolescents’ NSSI. Schools, families and society should pay more attention to the rational use of the Internet for adolescents. Secondly, this study found that loneliness is an important “bridge” linking IA to NSSI. Therefore, interventions that focus on alleviating loneliness in adolescents may be a potential strategy for preventing NSSI. Finally, the tests of our moderated mediation model contribute to the literature by providing evidence in support of the emotional regulation process model and the cognitive emotional model of NSSI. We found that cognitive reappraisal, as an individual difference, could explain the heterogeneity of the relationship between IA and NSS in adolescents. Thus, improving adolescents’ skills in cognitive reappraisal may be particularly useful in the treatment of NSSI (34). This model provides new information on the relationship between IA and NSSI in adolescents and offers potential ways to prevent the harmful consequences of IA on adolescents.

## Data availability statement

The raw data supporting the conclusions of this article will be made available by the authors, without undue reservation.

## Ethics statement

The studies involving humans were approved by Affiliated WuTaiShan Hospital of Medical College of Yangzhou University before the survey (Reference number: WTSLL20222009). The studies were conducted in accordance with the local legislation and institutional requirements. Written informed consent for participation in this study was provided by the participants’ legal guardians/next of kin.

## Author contributions

NL: Writing – original draft, Methodology, Investigation, Formal analysis, Conceptualization. HL: Writing – review & editing, Supervision, Project administration. LW: Writing – review & editing, Supervision, Investigation, Data curation. JY: Writing – review & editing, Supervision, Investigation, Data curation. AP: Writing – review & editing, Project administration, Investigation, Data curation.
